# Thermodynamics of Superdiffusion Generated by Lévy–Wiener Fluctuating Forces

**DOI:** 10.3390/e20090658

**Published:** 2018-08-31

**Authors:** Łukasz Kuśmierz, Bartłomiej Dybiec, Ewa Gudowska-Nowak

**Affiliations:** 1 Laboratory for Neural Computation and Adaptation, RIKEN Center for Brain Science, 2-1 Hirosawa, Wako, Saitama 351-0198, Japan; 2 Marian Smoluchowski Institute of Physics and Mark Kac Complex Systems Research Center, Jagiellonian University, ul. S. Łojasiewicza 11, 30-348 Kraków, Poland

**Keywords:** nonequilibrium and irreversible thermodynamics, fluctuation phenomena, random walks and Lévy flights, 05.70.Ln, 05.40.-a, 05.40.Fb

## Abstract

Scale free Lévy motion is a generalized analogue of the Wiener process. Its time derivative extends the notion of “white noise” to non-Gaussian noise sources, and as such, it has been widely used to model natural signal variations described by an overdamped Langevin stochastic differential equation. Here, we consider the dynamics of an archetypal model: a Brownian-like particle is driven by external forces, and noise is represented by uncorrelated Lévy fluctuations. An unperturbed system of that form eventually attains a steady state which is uniquely determined by the set of parameter values. We show that the analyzed Markov process with the stability index α<2 violates the detailed balance, i.e., its stationary state is quantified by a stationary probability density and nonvanishing current. We discuss consequences of the non-Gibbsian character of the stationary state of the system and its impact on the general form of the fluctuation–dissipation theorem derived for weak external forcing.

## 1. Introduction: Lévy Stable Distributions and Lévy Stable Processes

The normal distribution plays a special role in statistics and physics. This is due to the abundance of the observations displaying Gaussian fluctuations which is explained by the central limit theorem. Additionally, the Gaussian assumption in tandem with the Markov property, in many cases, makes calculations more manageable. Therefore, Gaussian white noise is used as an archetypal process modeling complex interactions of a test particle with its environment. This approximation works perfectly well when interactions are independent and bounded. Both these assumptions can be violated resulting in more general non-Markovian and non-Gaussian processes. Also, vast experimental evidence indicates that fluctuations can be of a more general, non-Gaussian type.

Many collective, complex physical systems are characterized by fluctuations strongly deviating from the “canonical” Gaussian description, often categorized by diverging mean and variance. In terms of the probability calculus, distributions governing this type of behavior are frequently identified with so called Lévy α-stable laws [1], where “stability” signifies that the product of characteristic functions of two independent such laws results in a characteristic function of another variable of the same type. Moreover, according to the generalized central limit theorem [2], these distributions appear as limits of sums of independent and identically distributed random variables with divergent variance, i.e., random variables with probability density functions behaving as the power law $1/{\left|x\right|}^{\alpha +1}$ for large |x| values.

Lévy statistics are omnipresent and have been used as valuable models of various data sets—they have been detected in the critical state and in self-organized criticality phenomena. They correctly fit empirical distributions of extreme events, like avalanches and earthquakes, and describe the broadening of spectral lines in plasma, the distribution of gravitational force between randomly localized stars, and relaxation phenomena in disordered systems well [3]. A generalization of Gaussian white noise to its non-Gaussian, Lévy stable counterpart serves as a model of impulsive, large scale variations observed, e.g., in turbulent heat flow [4] and solar flare fluctuations [5,6], hole transport in semiconductors [7], transmission of light in inhomogeneous materials [8], and anomalous diffusive transport [9,10]. Apart from financial mathematics where the Lévy fluctuations became an attractive model for price variation [11], there is also accumulating evidence from biological experiments showing that production of mRNA and proteins occurs in a pulsatile manner and creates non-Gaussian noise in individual cells [12,13,14]. Those bursty events result in high transcriptional activity followed by long periods of inactivity and are characterized by heavy tailed distributions and Lévy-like statistics. Moreover, the cytoplasmatic mechanical activity has been documented to be far from equilibrium [15], and the total intensity of cytoskeletal noise has been estimated to exceed the level of thermal noise ($k_{B}T$). Since the intrinsic stochastic excitations may play a crucial role in transcriptional regulatory systems [14], it could well be that non-Gaussian Lévy noise should be a proper model of choice for underlying fluctuations in biological systems.

Over the past years, stochastic differential equations with non-Gaussian Lévy noises have gained a lot of interest in regard to modeling the DNA-target search for binding sites [16], active transport within cells [17], and search strategies [18,19,20,21,22,23,24]. In all of those problems, the primary focus is on proposing a stochastic model to address the issues of relaxation and kinetics of the system under investigation. The paradigmatic choice of preference is usually the Langevin equation, for which stochastic energetics have been defined under the action of Gaussian white noise [25,26]. It thus seems plausible to deeply explore the possibilities and limitations of the Langevin approach to study systems with a more general form of fluctuating forces. Before addressing this point, we briefly review the properties of α-stable laws [1,27,28,29,30,31,32] and discuss the profoundly nonequilibrium properties of Lévy-stable noises.

**Definition** **1.** *Let $X_{1}$ and $X_{2}$ be independent identically distributed random variables. The distribution they share is said to be stable if*

(1) $$\forall a,b>0\;\exists c,d\in R\;\forall x:F_{aX_{1}+bX_{2}}\left(x\right)=F_{cX_{1}+d}\left(x\right),$$

*where $F_{X}$ stands for the distribution function of X. If d=0 for every pair (a,b), then the distribution is said to be strictly stable.*

The family of all distributions which satisfies the requirement of stability was constructed by Paul Lévy [27]. These distributions are referred to as α-stable distributions or Lévy α-stable distributions.

**Definition** **2.** *Let α∈(0,2] and β∈[−1,1]. A random variable ($S_{\left(\alpha ,\beta \right)}$) is called α-stable if its characteristic function is given by the formula*

(2) $$\ln\Phi_{S_{\left(\alpha ,\beta \right)}}\left(k\right)=\left\{\begin{array}{cc} -{\left|k\right|}^{\alpha }\sigma^{\alpha }\left(1-i\beta \mathrm{sgn}\left(k\right)\tan\frac{\pi \alpha }{2}\right)+ik\mu & \mathrm{for}\;\;\;\alpha \neq 1\\ -|k|\sigma \left(1+i\beta \mathrm{sgn}\left(k\right)\frac{2}{\pi }\log\left|k\right|\right)+ik\mu & \mathrm{for}\;\;\;\alpha =1 \end{array}\right.,$$

*where sgn(·) is the sign function. In the special case of β=0, this formula simplifies to*

(3) $$\ln\Phi_{S_{\left(\alpha ,0\right)}}\left(k\right)=-{\left|k\right|}^{\alpha }\sigma^{\alpha }+ik\mu ,$$

*and the corresponding $S_{\left(\alpha ,0\right)}$ is called a symmetric α-stable random variable.*

In accordance with Samorodnitsky [1], we recall here a representation of standard α-stable distributions with the following parameters: α (identified as a characteristic exponent or a stability index), β (responsible for the skewness of the probability law), and σ—called the scale parameter that control thes overall distribution width. Finally, μ is the location parameter. The stability index α describes the asymptotic (i.e., large *x*) behavior of the density function ($p\left(x\right)=\frac{d}{dx}F_{X}\left(x\right)$), which for the symmetric case with α<2 assumes the form

(4) $$p\left(x\right)\propto \frac{\sigma^{\alpha }\sin\left(\frac{\pi \alpha }{2}\right)\Gamma \left(\alpha +1\right)/\pi }{{\left|x\right|}^{\alpha +1}}.$$

Note that for α<2, any α-stable density is characterized by the diverging standard deviation. Moreover, for α<1, the mean value also diverges.

**Definition** **3.** *A stochastic process ({X(t),t⩾0}) is known as α-stable Lévy motion (Lévy flight) if X(0)=0 and the process has independent increments ΔX, distributed according to the α-stable law $\Delta X\equiv X\left(t\right)-X\left(s\right)∼S_{\left(\alpha ,\beta \right)}\left({\left(t-s\right)}^{1/\alpha }\right)$ for any t,s, such that 0⩽s⩽t⩽∞.*

**Figure 1:**
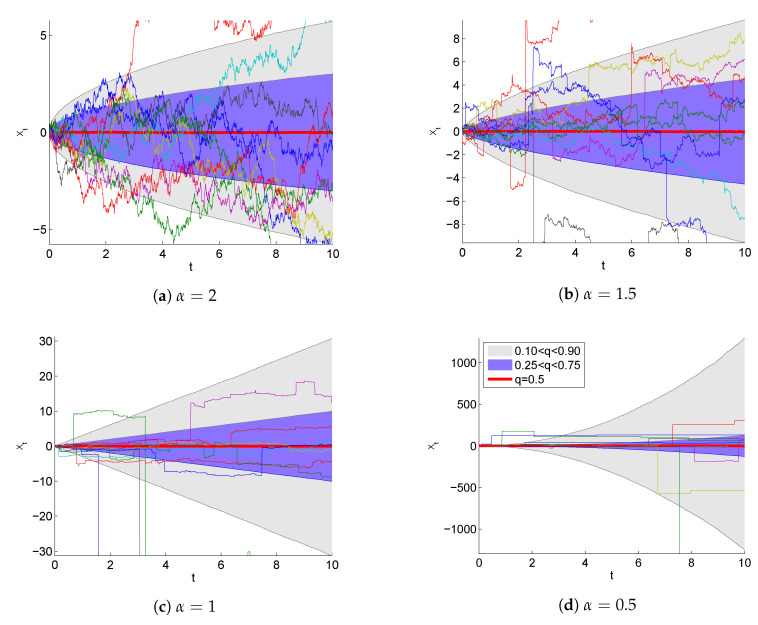
Visualizations of 1-dimensional α-stable processes for α=(2;1.5;1;0.5). Along with ten exemplary sample trajectories, the median (red) and quantile areas (gray and violet) are plotted. The quantiles were obtained from $10^{5}$ sample trajectories integrated with the Euler method (Δt=0.01).

Following this definition, the formal time derivative of the symmetric α-stable motion defines a generalization of the Gaussian white noise, i.e., a symmetric Markov α-stable noise which turns into a standard Gaussian form for α=2. Lévy flights thus extend the Brownian motion paradigm to self-similar motions ({X(ct),t⩾0} and $\left\{c^{1/\alpha }X\left(t\right),t⩾0\right\}$ have the same distributions) with uncorrelated random steps. However, unlike standard Brownian motions for which the mean-squared displacement (MSD) grows in time ($\langle \Delta X^{2}\left(t\right)\rangle \propto t$), the dispersion of the position in the Lévy motion diverges, and the width of the resulting asymptotic Lévy (super)-diffusion must be characterized by some fractional moments [30] or the interquantile distance (see Figure 1). This fact indicates that the Lévy flight, i.e., the stochastic process defined by Equation (5), displays some non-physical properties, which are especially visible for α<2. Due to the heavy tails of α-stable densities, with α<2, there is a significant probability of extremely long jumps leading to the divergence of variance of Lévy flights. The resulting infinite propagation velocity can be eliminated through the spatio-temporal coupling of jump lengths and associated waiting or travel times resulting in so-called Lévy walks [33,34]. The non-Markovian character of Lévy walks, however, impedes the stochastic thermodynamics analysis of these systems. Here, we focus on the study of fluctuation dissipation relations for Lévy flights and direct the interested reader to the further discussion of differences and analogies between Lévy walks and Lévy flights in Ref. [35].

Lévy flights and Lévy fluctuations are ubiquitous in nature. They occur in turbulent flows [36], incoherent radiation trapping [37] diffusion of particles in random media [38], the spreading of epidemics and human travel behavior [39], in economic and paleoclimatic time series [40,41] and in random movements of the cell cytoskeleton generated by motor proteins [42]. The evidence for bursty, Lévy flight-like behavior in human cognition retrieval from semantic memory and mental searches have been recently documented [18,19,43]. Despite their omnipresence, the thermodynamics of non-Gaussian stable fluctuations poses several objectives [44,45] beyond the standard description typical for Gaussian cases. In the forthcoming brief resume, we comment on some unusual properties of Lévy fluctuations and their thermodynamic consequences.

## 2. Lévy Flights and Detailed Balance

Let ${\left(X_{t}\right)}_{t⩾0}$ be a stochastic process that is solved by the following Langevin equation:

(5) $$dX_{t}=-aX_{t}dt+dS_{t}^{\left(\alpha \right)},$$

where $dS_{t}^{\left(\alpha \right)}$ stands for increments of the Lévy–Wiener process (Lévy motion) defined above. Equation (5) describes the overdamped motion of the random walker in the static harmonic potential subjected to the action of the α-stable (Lévy) noise. Since the system is overdamped, its state is fully determined by position x(t) which is a random variable. Alternatively, the probability density function (PDF) of the process p(x,t) obeys [30,45,46,47] the corresponding fractional Smoluchowski–Fokker–Planck Equation (SFPE):

(6) $$\partial_{t}p\left(x,t|x_{0}\right)=\sigma_{0}^{\alpha }\frac{\partial^{\alpha }}{\partial {\left|x\right|}^{\alpha }}p\left(x,t|x_{0}\right)+a\frac{\partial }{\partial x}\left(xp(x,t|x_{0})\right),$$

which is conditioned on the value of the stochastic process at time $t_{0}=0$ (the process is time homogeneous). Parameter *a* sets the time constant of the deterministic part of the system. For the sake of brevity, in the following, we put a=1. The fractional operator $\frac{\partial^{\alpha }}{{\partial |x|}^{\alpha }}$ with 0<α⩽2 stands for the Riesz fractional derivative, defined by the Fourier transform [28,29]: $F_{k}\left(\frac{\partial^{\alpha }f\left(x\right)}{{\partial |x|}^{\alpha }}\right)=-{\left|k\right|}^{\alpha }F_{k}\left(f\left(x\right)\right).$ Accordingly, in the (fractional) diffusion equation (Equation (6)), the term $\sigma_{0}^{\alpha }$ can be interpreted as the generalized diffusion constant [3,48], where *σ*_0_ is the scale parameter that characterizes the underlying Lévy noise in the overdamped Langevin Equation (5). The generalized diffusion coefficient is related to the distribution asymptotics (see Equation (4) and Ref. [48]). Please note, that in situations when finite propagation velocity is assumed, one can calculate the constrained or “moving” mean squared displacement [49] which differs from the generalized diffusion coefficient (compare with Refs. [48,49]). The solution of this SFPE is given by

(7) $$p\left(x,t|x_{0}\right)=\frac{1}{\sigma (t)}S_{\alpha }\left(\frac{x-\mu (t)}{\sigma (t)}\right),$$

where $S_{\alpha }\left(\cdot \right)$ is the standard symmetric α-stable distribution whose Fourier transform is

(8) $$F\left\{S_{\alpha }\right\}\left(k\right)=e^{-{\left|k\right|}^{\alpha }}.$$

The time-dependent location (μ(t)) and scale (σ(t)) parameters read

(9) $$\mu \left(t\right)=x_{0}e^{-t}$$

and

(10) $$\sigma \left(t\right)=\sigma_{0}{\left(\frac{1-e^{-\alpha t}}{\alpha }\right)}^{1/\alpha }.$$

The unique stationary state is denoted $p_{s}\left(x\right)=\lim_{t\to \infty }p_{s}\left(x,t|x_{0}\right)$. The stationary state is given by the α-stable density with a different scale parameter than in the noise term, i.e., $\sigma_{\infty }=\sigma_{0}/\alpha^{1/\alpha }$, which is the t→∞ limit of Equation (10).

A dynamic system driven by fluctuating forces (Equation (5)) is said to be microscopically reversible if the probability of its trajectory and its time reversal are identical, so that the work imposed by external forcing equals the change in free energy [50,51]. Here, we examine the flow of probability between the states and show that the relative probabilities of trajectories in the Lévy–Wiener process do not fulfill the detailed balance condition.

**Definition** **4.** *We say that there is a detailed balance if the following condition is met:*

(11) $$\forall_{x,y\in R,t>0}:p\left(x,t|y\right)p_{s}\left(y\right)=p\left(y,t|x\right)p_{s}\left(x\right).$$

**Theorem** **1.** *The detailed balance holds for the solution of (5) if α=2.*

**Proof.**  The following calculations show that the detailed balance condition (11) is trivially fulfilled for α=2. (For the sake of clarity we have assumed $\sigma_{0}=1/\sqrt{2}$ here which corresponds to the standard normal density N(0,1), see Equation (2). It is trivial to prove that a change in the parameter does not change the result (Theorem 1)).

$$\begin{array}{cc} \forall_{x,y\in R,t>0}: & \ln\left(\frac{p\left(x,t|y\right)p_{s}\left(y\right)}{p\left(y,t|x\right)p_{s}\left(x\right)}\right)=\ln\left(\frac{\exp\left(-\frac{{\left(x-ye^{-t}\right)}^{2}}{2(1-e^{-2t})}\right)\exp\left(-\frac{y^{2}}{2}\right)}{\exp\left(-\frac{{\left(y-xe^{-t}\right)}^{2}}{2(1-e^{-2t})}\right)\exp\left(-\frac{x^{2}}{2}\right)}\right)=\\ = & -\frac{{\left(x-ye^{-t}\right)}^{2}-{\left(y-xe^{-t}\right)}^{2}+\left(1-e^{-2t}\right)y^{2}-\left(1-e^{-2t}\right)x^{2}}{2(1-e^{-2t})}=0. \end{array}$$

Now, let us turn to the case with α<2. From (11) it follows that, in particular,

(12) $$g\left(x,t\right)\equiv \frac{p\left(x,t|0\right)p_{s}\left(0\right)}{p\left(0,t|x\right)p_{s}\left(x\right)}=1$$

and thus

(13) $$h\left(x\right)\equiv \lim_{t\to 0}g\left(x,t\right)=1.$$

Conditions (12) and (13) are necessary, but not sufficient, for the detailed balance to hold. For α<2 and |x|>0, we have (note that $\lim_{t\to 0}\sigma \left(t\right)=0$)

(14) $$\lim_{t\to 0}\frac{p(x,t|0)}{p(0,t|x)}=\lim_{t\to 0}\frac{S_{\alpha }\left(\frac{x}{\sigma (t)}\right)}{S_{\alpha }\left(-\frac{\mu (t)}{\sigma (t)}\right)}=\lim_{t\to 0}{\left(\frac{\sigma (t)}{\left|x\right|}\right)}^{1+\alpha }{\left(\frac{\left|x\right|e^{-t}}{\sigma (t)}\right)}^{1+\alpha }=1.$$

**Figure 2:**
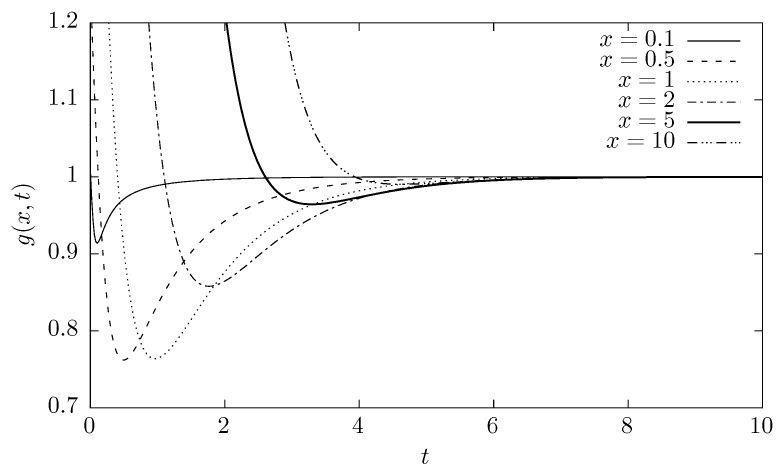
The ratio g(x,t) (see Equation (12)) for α=1 and various values of *x*.

Intuitively, this means that for very short times, the propagator does not depend on the deterministic force felt by the particle (see Equation (5)). By plugging this into (12) and (13), we arrive at (x>0):

(15) $$h\left(x\right)=\frac{S_{\alpha }\left(0\right)}{S_{\alpha }\left(x\right)}>1,$$

which shows that the detailed balance does not hold for α<2 (note that for a symmetric stable variable, h(x) takes its maximum value at x=0 and decays beyond this value). This observation can be easily checked for the Cauchy–Wiener process (α=1) whose propagator ($p(x,t|x_{0})$) satisfying Equation (6) reads

(16) $$p\left(x,t|x_{0}\right)=\frac{\sigma (t)}{\pi }\frac{1}{{\left(x-\mu \left(t\right)\right)}^{2}+\sigma^{2}\left(t\right)}$$

with $\sigma \left(t\right)=\sigma_{0}\left(1-e^{-t}\right)$ and $\mu \left(t\right)=e^{-t}x_{0}$. Accordingly, Figure 2 displays the sample ratios (g(x,t)) for this case and various values of x. For small t and moderate values of x, clear deviations from g(x,t)=1 are visible. A similar result was obtained a particle subjected to Cauchy (α=1) white noise in the parabolic potential well in Ref. [52], where the nonzero flow of probability between the segments of the well was demonstrated. ☐

Note that for a system governed by Gaussian fluctuations ($dS_{t}^{\left(2\right)}$), the corresponding SFPE takes on the form of the conservation law of the probability ($\partial_{t}p\left(x,t|x_{0}\right)+\nabla J\left(x,t\right)=0$) with the current ($J\left(x,t\right)=-xp\left(x,t|x_{0}\right)-\sigma_{0}^{2}\partial_{x}p\left(x,t|x_{0}\right)$) and *σ*_0_, representing the intensity of the noise at the level of the Langevin equation [53]. At steady state, the divergence of the flow has to vanish [54]. For a trivial case (J≡0), this implies that $-xp_{s}\left(x\right)-\sigma_{0}^{2}\partial_{x}p_{s}\left(x\right)=0$, and the driving force can be written as

(17) $$-x=\frac{\sigma_{0}^{2}\frac{\partial }{\partial x}p_{s}\left(x\right)}{p_{s}\left(x\right)}=-\sigma_{0}^{2}\frac{\partial }{\partial x}\left(-\log p_{s}\left(x\right)\right)=-\frac{\partial }{\partial x}U\left(x\right)$$

with a potential U(x)

(18) $$U\left(x\right)=-\sigma_{0}^{2}\left(\ln p_{s}\left(x\right)+\ln Z\right).$$

In other words, under Gaussian fluctuations, the detailed balance condition (J≡0) is equivalent to the requirement that at equilibrium, the Boltzmann relationship between the weight of the state (equilibrium probability) and underlying potential surface exists (clearly, this condition does not need to be satisfied for general nonequilibrium systems [54]).

Condition J≡0 implies a further thermodynamic relationship [25,26] between the probability density function and the relative or distance entropy that measures deviation from the stationary state ($p_{s}\left(x\right)$):

(19) $$\sigma_{0}^{2}\int p\left(x,t\right)\ln\frac{p(x,t)}{p_{s}\left(x\right)}dx=\langle U\left(x\right)\rangle -\sigma_{0}^{2}\left(-\int p\left(x,t\right)\ln p\left(x,t\right)dx\right)+\sigma_{0}^{2}\ln Z,$$

where $Z\equiv \int \exp(-U\left(x\right)/\sigma_{0}^{2})dx$ is the partition function, $F\equiv -\sigma^{2}\ln Z$ stands for the free energy function, and the second term on the RHS denotes the Shannon entropy. If the strength of fluctuations ($\sigma^{2}$) can be related to the ambient temperature *T*, the left-hand-side of the expression above achieves a simple interpretation of the instantaneous free energy of the stochastic system [25,50] at hand.

Notably, the relative entropy (or the instantaneous free energy of the system) defined above plays a role in the Lyapunov function (H-functional) [44] for a general class of stochastic Markovian systems described by Equations (5) and (6). Through an analogy to the Gibbs equilibrium state (cf. Equations (18) and (19)), it is thus tempting to interpret the functional $\Phi \equiv -\ln p_{s}\left(x\right)$ as a nonequilibrium pseudo-potential [25,50,52,55,56] from which the equivalents of “internal” and “free” energies of the system can be identified as in Equation (19) above. This approach yields Φ≠F(x)m and is incompatible with the requirement that the reference (stationary) state should correspond to the Gibbs equilibrium with $p_{s}\left(x\right)$ fulfilling Equation (18). Indeed, the issue of thermalization or equilibration of Lévy flights can be only addressed by predefined confinement of the motion in properly “tailored” potentials [31,32,44,57,58,59], or otherwise described by nonextensive Tsallis’ thermodynamics [60]. There are also known “non-Langevin” scenarios [61,62] based on a master equation that result in Levy flights fulfilling a detailed balance with the stationary state of the Boltzmann–Gibbs type.

The scaling properties (Equation (7)) of the time dependent density p(x,t) result for the general case in dynamic (Shannon) entropy (cf. Equation (17)) taking on the form

(20) $$S\left[p\left(x,t\right)\right]=-\int S_{\alpha }\left(z\right)\ln S_{\alpha }\left(z\right)dz+\ln\sigma \left(t\right),\;z=x/\sigma \left(t\right),$$

thus indicating the entropy production rate:

(21) $$\frac{dS[p(x,t)]}{dt}=\frac{\dot{\sigma }\left(t\right)}{\sigma (t)}.$$

In the case of the free Lévy flights described by the stability index α, the above rate formula results in $\frac{d}{dt}S\left[p\left(x,t\right)\right]\propto {\left(\alpha t\right)}^{-1}$, i.e., the entropy production rate is positive, decreases for increasing α, and attains a minimal value for α=2, corresponding to generic Gaussian fluctuations, typical for states close to equilibrium [63,64]. Similarly, for Lévy flights in the quadratic potential, the evaluation of Equation (21) yields $\frac{d}{dt}S\left[p\left(x,t\right)\right]=\frac{1}{e^{\alpha t}-1}$ which coincides with the rate of a free Lévy process for short times (t≪1).

These significant differences in the statistical properties of systems driven by non-Gaussian Lévy fluctuations, and, in particular, divergence of the second moment, imply the lack of a simple Einstein’s fluctuation–dissipation relationship between fluctuation strength and the magnitude of dissipation. In a forthcoming section, we review the linear relaxation theory based on the identity derived by Hatano and Sasa [65] for dynamic Markov systems and present an extension of the generalized fluctuation–dissipation theorem to the nonequilibrium system subjected to thermal (Gaussian noise) and nonthermal (external Cauchy noise) fluctuations.

## 3. Linear Response and Fluctuation–Dissipation Relationship under Lévy Noise

Relation between externally induced and spontaneous fluctuations in systems close to equilibrium is described by the concept of the response function [66,67]. Linear response of a generic observable X(t) measured at time *t* to small perturbations f(t) and the correlation between unperturbed observable is characterized by the susceptibility measured in the reference, unperturbed state:

(22) $$\chi \left(t,t^{\prime }\right)\equiv -\frac{1}{k_{B}T}\frac{d}{dt}\langle \delta X\left(t\right)\delta X\left(t^{\prime }\right)\rangle {|}_{f=0}$$

where

(23) $$\delta X\left(t\right)=\langle X\left(t\right)\rangle -\langle X\left(t^{\prime }\right)\rangle {|}_{f=0}\propto \int_{0}^{t}\chi \left(t-t^{\prime }\right)\delta \left(f\left(t^{\prime }\right)\right)dt^{\prime }.$$

As a result, the celebrated Green–Kubo fluctuation–dissipation theorem Equation (22) links relaxation properties of the system to correlations of spontaneous fluctuations around the equilibrium state.

For systems described by the Hamiltonian H(x) and manipulated by weak forces f(t), the generic observable X(t) is identified as a “conjugate variable” in the Onsager’s theory and can be interpreted as the fluctuation of the quantity $\frac{\partial H(x,f)}{\partial f}{|}_{f=0}$, i.e.,

(24) $$X\left(x\right)=\frac{1}{k_{B}T}\left[\frac{\partial H(x,f)}{\partial f}-\langle \frac{\partial H}{\partial f}{|}_{f=0}\rangle \right]=\frac{1}{k_{B}T}\frac{\partial [H(x,f)-F(f)]}{\partial f}{|}_{f=0}.$$

Here, in line with Equations (18) and (19), the free energy is $F=-\sigma_{0}^{2}\ln Z=-k_{B}T\ln Z=-k_{B}T\ln\int dxZ^{-1}\delta \left(H\left(x\right)-U\left(x\right)\right)e^{-\frac{H}{k_{B}T}}$.

Onsager’s theory and Green–Kubo relationships are grounded on the expansion around equilibrium states, which restricts their applicability to systems close to equilibrium. However, the generalization of the fluctuation–dissipation theorem to out of equilibrium systems has been established in a number of cases [65,68,69]. In their seminal paper from 2001, Hatano and Sasa [65] proposed an identity for Markov dynamic systems evolving between two steady states. The identity has been further explored [55,68] to derive the general form of the fluctuation–dissipation theorem for non-energy conserving dynamics. In what follows, we discuss an extension of this formalism to the generalized fluctuation–dissipation theorem for systems away from equilibrium and under the action of non-equilibrated environment (heat bath). We model interactions of the particle with the reservoir by assuming that there are two statistically independent sources of noise affecting the particle: a white Gaussian noise and a white Cauchy noise. The latter introduces large impulses, leading, effectively, to infinite moments of the position of the particle and also, infinite average energy. Accordingly, the statistical temperature of the system cannot be properly defined, i.e., the system is out of equilibrium even in its stationary state. Yet, despite those peculiarities, by properly identifying variables conjugated with external perturbations, the generalized form of the fluctuation–dissipation theorem can be re-established and the validity of linear response to perturbations can be verified.

We focus our attention on the system described the following Langevin equation

(25) $$dX_{t}=-aX_{t}dt+f\left(t\right)dt+dS_{t}^{\left(2\right)}+dS_{t}^{\left(1\right)},$$

where ${\left(S_{t}^{\left(2\right)}\right)}_{t⩾0}$, and ${\left(S_{t}^{\left(1\right)}\right)}_{t⩾0}$ denote Wiener and symmetric Cauchy processes, respectively, with the scale parameters $\sigma^{\left(2\right)}=\sigma_{0}$ and $\sigma^{\left(1\right)}=\gamma_{0}$ standing for noise magnitudes and f(t) defining the external deterministic force field. Since the Langevin Equation (25) is linear, its solution depends linearly on two statistically independent noises and the corresponding probability distribution function (p(x,t)) of the dynamic variable (x(t)) attains the convoluted form of two Lévy PDFs with the stability indices α=1 and α=2. The corresponding characteristic function is expressed by a product

(26) $$\hat{p}\left(k,t\right)=e^{ik\mu \left(t\right)-\sigma^{2}\left(t\right){\left|k\right|}^{2}-\gamma \left(t\right)\left|k\right|},$$

and fulfills [56,70,71] the generalized Smoluchowski–Fokker–Planck equation

(27) $$\frac{\partial \hat{p}\left(k,t\right)}{\partial t}=-ak\frac{\partial }{\partial k}\hat{p}\left(k,t\right)+ik\left[\mu \left(t\right)+f\left(t\right)\right]\hat{p}\left(k,t\right)-\sigma_{0}^{2}{\left|k\right|}^{2}\hat{p}\left(k,t\right)-\gamma_{0}\left|k\right|\hat{p}\left(k,t\right).$$

The Lévy jump statistics entering dynamics through the noise term $S_{t}^{\left(1\right)}$ lead to an inherently nonequilibrium situation (note that due to the presence of the Cauchy noise, the expected value ($E[X_{t}]$) does not exist for $\gamma_{0}\neq 0$), and the overall probability distribution of the process deviates from the Gibbs–Boltzmann form, exhibiting bulk Gaussian part and asymptotic heavy tails. Inserting Equation (26) into the Smoluchowski–Fokker–Planck Equation (27) yields evolution equations for the scale and location parameters:

(28) $$\begin{array}{c} \dot{\mu }\left(t\right)=-a\mu \left(t\right)+f\left(t\right)\\ \dot{\gamma }\left(t\right)=\gamma_{0}-a\gamma \left(t\right)\\ \frac{d\sigma^{2}\left(t\right)}{dt}=\sigma_{0}^{2}-2a\sigma^{2}\left(t\right) \end{array}$$

where we assume μ(0)=0 at time t=0. For a constant force (f(t)=f), the long time limit of these equations gives the stationary parameters $\lim_{t\to \infty }\mu \left(t\right)=f/a$, $\lim_{t\to \infty }\gamma \left(t\right)=\gamma_{0}/a$, $\lim_{t\to \infty }\sigma^{2}\left(t\right)=\sigma_{0}^{2}/\left(2a\right)$ characterizing a non-equilibrium steady state $p_{s}\left(x,f\right)=\lim_{t\to \infty }p_{X_{t}}\left(x\right)$ of the system.

We further assume that the system is initially prepared in this stationary state. The question we are going to address is whether the generalized form of the fluctuation–dissipation theorem can be used here to test the linear response of the system to external drivings. This problem is addressed below in the framework of stochastic thermodynamics [25,26,50,52,53,54].

In line with Onsager’s theory, we first introduce a non-equilibrium pseudo-potential [55,56] $\Phi \left(x,f\right)=-\ln p_{s}\left(x,f\right)$ and use it to define a variable $X_{GC}$ that is conjugated to the perturbation *f*:

(29) $$X_{GC}\equiv -\frac{\partial \ln p_{s}\left(x\right)}{\partial f}{|}_{f=0}={\left.\frac{\partial \Phi (X_{t},f)}{\partial f}\right|}_{f=0}=-\frac{1}{a}{\left.\frac{\partial \Phi (x,0)}{\partial x}\right|}_{x=X_{t}},$$

where

(30) $$p_{s}\left(x\right)=\frac{1}{2\sqrt{\pi }\sigma_{\infty }}ℜ\left(w\left(\frac{-x+i\gamma_{\infty }}{2\sigma_{\infty }}\right)\right).$$

$w\left(x\right)=e^{-x^{2}}\mathrm{erfc}\left(-ix\right)$ is the complex error function [1,29], and the second equality in Equation (29) follows from the linearity of Equation (25). Thus, the conjugate variable $X_{GC}$ defines a new stochastic process related to the original $\left\{X_{t},t⩾0\right\}$ by a nontrivial transformation:

(31) $$X_{GC}=-\frac{x}{2\sigma_{\infty }^{2}a}-\frac{\gamma_{\infty }}{2\sigma_{\infty }^{2}a}\frac{ℑ\left(w(\frac{-x+i\gamma_{\infty }}{2\sigma_{\infty }})\right)}{ℜ\left(w(\frac{-x+i\gamma_{\infty }}{2\sigma_{\infty }})\right)}.$$

**Figure 3:**
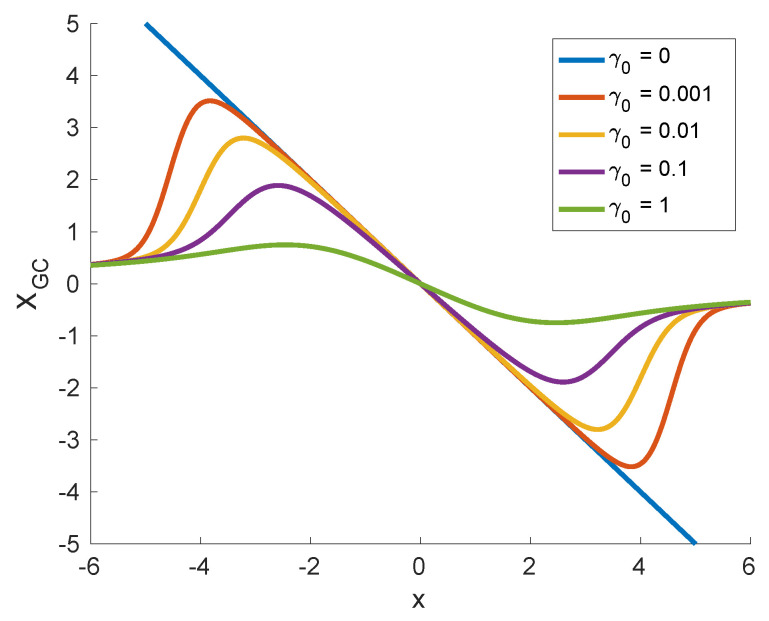
The nonlinear transformation defining the conjugate variable (29) for $\sigma_{0}=1$, a=1 and different values of $\gamma_{0}$. Note that $X_{GC}\left(x\right)$ is nonmonotonic for $\gamma_{0}>0$.

In Figure 3, the functional dependence ($X_{GC}\left(x\right)$) is displayed for $\sigma_{0}=1$, a=1 and several different values of the Cauchy noise strength ($\gamma_{0}$). In contrast to the pure Gaussian case (cf. Equations (26) and (29) with γ(t)≡0), when the conjugate variable reads $X_{G}=-2x/\sigma_{0}^{2}$, the variable $X_{GC}$ is a nonmonotonic function of *x*. This is related to the heavy tails of the noise distribution and not to the mixing of two types of noise, as can be seen from the form of the conjugate variable in the case of $\sigma_{0}=0,a=1$ [55,72]

(32) $$X_{C}\left(x\right)=-\frac{2x}{\gamma_{0}^{2}+x^{2}}.$$

The fluctuation–dissipation theorem (or its generalized form) is expected to link the autocorrelation function of the conjugate variable $\langle X_{GC}\left(t\right)X_{GC}\left(0\right)\rangle$ to a generalized susceptibility

(33) $$\chi \left(t\right)=\frac{d}{dt}\langle X_{GC}\left(t\right)X_{GC}\left(0\right)\rangle {|}_{f=0},$$

where the averaging is performed over the stationary (unperturbed f=0) state. The expected response of the conjugate variable to the perturbation f(t) can be then either determined exactly from

(34) $$\langle X_{GC}\left(t\right)\rangle =\int_{-\infty }^{\infty }p_{X_{t}}\left(x,t\right)X_{GC}\left(x\right)dx,$$

where $p_{X_{t}}\left(x,t\right)$ is calculated including f(t) and under stationary initial conditions or otherwise, estimated from the linear response function

(35) $${\langle X_{GC}\left(t\right)\rangle }_{LR}=\int_{0}^{t}\chi \left(t-s\right)f\left(s\right)ds.$$

**Figure 4:**
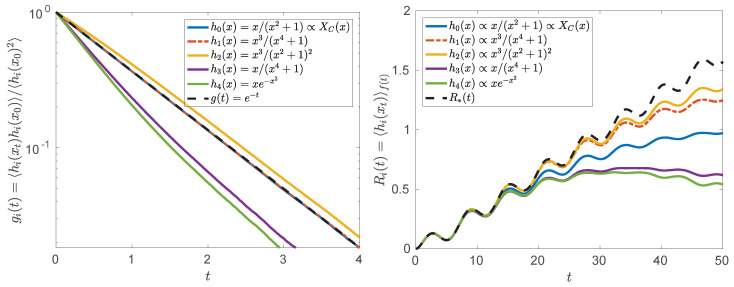
(**Left**) Autocorrelation functions of different nonlinear transformations of process $X_{t}$, which evolves according to Equation (25) with f(t)=0. (**Right**) Average reactions of various observables ($h_{i}\left(x_{t}\right)$) to external driving of the form f(t)=sint/10+t/30, as tested by a linear response. The function $R_{*}\left(t\right)$ represents the linear response ($R_{*}\left(t\right)=A\int_{0}^{t}e^{-s}f\left(s\right)ds$) with a constant *A* used to scale the data. All results were obtained by means of stochastic simulations with a=1, $\sigma_{0}=0$, $\gamma_{0}=1$, and the integration time step $\Delta t=10^{-2}$, averaged over $10^{7}$ independent realizations of the process.

The susceptibility and the corresponding linear response (LR) of the conjugate variable (35) can be easily derived analytically [55] if either γ(t)=0 or σ(t)=0. Remarkably, although the linear system with pure Cauchy noise (σ(t)=0,γ(t)≠0) has a nonlinear conjugate variable (32), its susceptibility is proportional to $e^{-t}$, as in the case of pure Gaussian noise ((σ(t)≠0,γ(t)=0)) with a linear conjugate variable. We confirmed that prediction with simulations (see Figure 4). In other cases χ(t) can be obtained by averaging over a large number of simulations of the unperturbed stochastic differential equation (SDE). Importantly, the simulations show that the combination of Gaussian and Cauchy noise (25) preserves the exponential time dependence of the susceptibility. This seems to be a general property of the susceptibility of linear systems driven by additive white noise and can be investigated by spectral decomposition of the fractional Fokker–Planck equation [73]. Similarly, exact responses according to Equation (34) can be obtained by means of stochastic simulations of the perturbed SDE.

**Figure 5:**
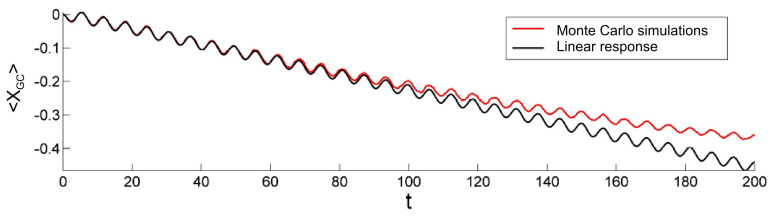
Response of the system to external driving f(t)=sin(t)/10+t/100 evaluated by Monte Carlo solution to the Langevin equation (red line) and compared with the result of the linear response theory (black line). The graphs represent the results obtained for constant scale parameters ($\sigma_{0}^{2}=1$ and $\gamma_{0}=1$). The deterministic relaxation time $a^{-1}$ was set to 1.

We performed tests of the generalized fluctuation–dissipation theorem by analyzing the response of the system to the sum of a small periodic and a linearly increasing force (f(t)). The integrals in Equation (35) were evaluated numerically and compared with the exact results of Equation (34) (see Figure 5). A comparison between the exact Equation (34) response and the one evaluated by means of the generalized fluctuation–dissipation theorem Equation (35) clearly indicates that the theory holds for small forcing (f(t)). This observation seems, at first sight, rather counterintuitive: Unlike standard Gaussian uncorrelated fluctuations, Lévy noises introduce bursty-like large jumps to the overall displacement ($dX_{t}$) causing divergence of the mean-squared displacement [45,52]. However, suitably chosen conjugate variable provided by the generalized fluctuation dissipation theorem (FDT) allow the response of the system to be analyzed using the concept of susceptibility (χ(t)). It should be noted that although the exact response can be derived using Equation (34) directly, the formulas are cumbersome and can be solved numerically in the simplest cases only. Therefore, the generalized FDT is a practical tool [55].

This is, however, not without a price. The conjugate variable used in the framework of FDT represents the change in the probability distribution of the system under the action of external disturbances. Close to equilibrium, such change is related to the energy absorbed by the system from external forcing. For non-equilibrium systems driven by Lévy fluctuations, this interpretation may be invalid. First, the conjugate variable is not just a displacement related to the external forcing (cf. Figure 3). Next, its proper identification requires knowledge of the stationary state which might be a demanding task for nonlinear systems. Last, but not least, divergence of the moments of dynamic variable *x* plague the system with infinite energy [72,74,75] (e.g., the potential energy of the system in the harmonic potential studied above).

We have checked whether the linear response function (35) can be used to predict responses of different nonlinear transformations (observables) of the dynamic variable, see Figure 4. To our surprise, some of the observables responded closer to the prediction of the linear response function than the conjugate variable. This suggests that the conjugate variable is not necessarily the best observable to monitor linear response and more detailed research is required to identify the conditions under which the linear response works.

## 4. Summary and Conclusions

We analyzed the peculiarities of jump-like Lévy noises with heavy tail statistics and demonstrated their distinctively different features from their Gaussian counterparts. For probability density functions generated by Langevin equation with non-Gaussian noise (Poisson shot noise or Lévy noises), violations of conventional steady states and transient fluctuation relations have been demonstrated [56,74,75,76], and the athermal character of non-Gaussian random forces has been investigated [77]. Here, we showed that despite the strong nonequilibrium character of Lévy flights, the generalized version of Onsager’s theory and fluctuation–dissipation theorem can be adapted to capture the dynamic responses of the system subjected to this type of noise. The linear response to external perturbation can be correctly predicted if the system is properly analyzed, i.e., one should transform the observables into the corresponding conjugate variables.

We predict that studying the fluctuations and linear responses of nonequilibrium systems with heavy-tailed noise will be useful for understanding active transport phenomena in biology. Lévy flights-like patterns have been observed in movement data of diverse organisms, including albatrosses [78], humans [79], honeybees [80], swarms of bacteria [81], and many others [82]. This is being commonly explained by the fact that this kind of behavior can constitute an advantageous random foraging strategy [20,21,24,83]. Although many models have been proposed [22,82,84], most of them apply to very specific cases and are yet to be tested experimentally. Therefore, the generative mechanisms behind Lévy patterns are still obscure. The linear response theory with nonlinear conjugate variables may be a fruitful way of looking at these systems, helping to identify the source of Lévy noise by applying perturbative interventions and inferring the underlying mechanisms.

The framework described in this paper is limited to white noise. Generalizations of the linear response theory to non-Markovian processes, such as subdiffusive continuous time random walks [47,48] and superdiffusive Lévy walks [33] are needed. Another interesting avenue for future research are systems with stochastic resetting [20,21,85,86], in which the particle is transported back to the initial position at random times. The stochastic thermodynamics of resetting has recently been analyzed [87]. However, it seems likely that the linear response cannot be directly applied in systems subject to stochastic resetting. For example, the nonequilibrium steady state of one-dimensional diffusion with stochastic resetting is given by the Laplace distribution, which leads to a noninformative conjugate variable that is proportional to the sign of the position.
